# Improving Outpatient Psychotherapy for Adults With Major Depressive and Anxiety Disorders Using Web-Based High-Frequency Monitoring and Feedback in Autosystemic Hypnotherapy: Protocol for a Two-Arm ABAB Crossed-Therapist Randomized Clinical Implementation Trial

**DOI:** 10.2196/78166

**Published:** 2026-01-07

**Authors:** Günter Schiepek, Stephanie Wackernagel

**Affiliations:** 1 Institute of Synergetics and Psychotherapy Research Paracelsus Medical University Salzburg Austria; 2 Faculty of Psychology and Education Ludwig-Maximilians-Universität München Munich Germany

**Keywords:** major depressive disorder, anxiety disorders, outpatient psychotherapy, autosystemic hypnotherapy, randomized clinical trial, routine process monitoring, high-frequency monitoring, synergetic navigation system, therapist effects, feedback effects, mobile phone

## Abstract

**Background:**

In recent years, routine outcome monitoring has been increasingly complemented by routine process monitoring in psychotherapy and other health care settings. Various approaches to therapy feedback exist, differing in assessment frequency, integration into the therapeutic process, and degree of personalization. In this study, we will use a procedure of high-frequency assessment through daily self-ratings, a standard process questionnaire, alongside a personalized questionnaire derived from case formulation, and frequent feedback interviews using visual diagrams to mirror the ongoing therapeutic processes.

**Objective:**

This study aims to investigate the effectiveness of combining routine process monitoring with hypno-psychotherapy (autosystemic hypnotherapy) by comparing it to autosystemic hypnotherapy without process feedback in the outpatient treatment of mood disorders. It also seeks to examine process-outcome relationships and mechanisms of change through high-frequency self-assessments and session-based feedback.

**Methods:**

This study is a randomized controlled trial with 2 arms, using within-therapist randomization (ABAB design) in outpatient psychotherapy. Participants are recruited offline via routine intake procedures. A total of 100 patients will be randomly assigned to one of the two conditions following a waiting period. The inclusion criterion is the existence of any mood disorder (major depressive disorder or anxiety disorder), assessed via a clinical interview. Each therapist treats patients in both conditions. Outcomes will be measured at 4 time points: after diagnosis confirmation, postwaiting period, posttreatment, and a 6-month follow-up. Primary and secondary outcomes, including symptom severity, will be assessed using questionnaires. Data collection also includes patient and therapist session evaluations using the Bern Patient and Therapist Session Questionnaire. In the feedback condition, therapists conduct frequent interviews using time-series data generated from daily self-assessments using the synergetic navigation system, including the Therapy Process Questionnaire and an individualized measure based on case conceptualization.

**Results:**

While this study is ongoing, the primary aim is to assess the effects of the feedback condition on therapeutic outcomes, including symptom reduction and patient motivation. This study will also explore how dynamic monitoring and feedback influence the therapeutic alliance and session-level improvements. It is expected that the feedback condition will lead to improvements in symptom severity and therapeutic engagement compared to the nonfeedback condition. Recruitment is ongoing, with 22 participants enrolled. The training of therapists and the data collection began in 2022. Data collection will end and study findings will be published in 2027. The German Society for Auto-Systemic Hypnotherapy is funding these training courses.

**Conclusions:**

This study combines effect and process measures within a feedback condition, compared to a nonfeedback condition. It incorporates dynamic process assessment to explore change mechanisms by analyzing patterns of time-series data and session ratings by patients and therapists. The approach provides insights into how continuous feedback and tailored monitoring influence therapeutic progress and outcomes.

**Trial Registration:**

OSF Registries osf.io/z2efy; https://doi.org/10.17605/OSF.IO/Z2EFY

**International Registered Report Identifier (IRRID):**

DERR1-10.2196/78166

## Introduction

### Effects of Therapy Monitoring and Feedback

For many years, monitoring and feedback procedures have been implemented in mental health care and in psychotherapy. Some researchers and practitioners identify these procedures as part of “good practice” [1] and even as the “most significant innovation in therapy practice within the past 20 years” [2]. Studies (case studies, implementation reports, and randomized controlled trials [RCTs]) tried to investigate the effects of monitoring and feedback in different mental health settings, applied to the treatment of many different diagnoses. Meta-analyses report small to medium overall effect sizes and showed that the feedback significantly reduces the odds of treatment dropout [3,4]. With respect to the effects, it has to be noted that the methodological quality of the studies is different and often poor [5], for example, pragmatic trials were realized with outcome assessments which were not independent of the process measures [6], and studies often are statistically underpowered (small, expected effect sizes require larger sample sizes) [7]. Other criteria concern the proof of using the feedback as intended—such as the manipulation check in an experiment—which, in case of not being taken for granted, may reduce the effect size of interventions compared to treatment as usual [8], and the implemented spectrum of outcome and progress measures [9].

To obtain a deeper insight into the mechanisms of feedback procedures, it should be considered that there are different approaches to realize it. Depending on the paradigmatic frame of the procedure, which can be linear and standardized or nonlinear [10,11], there may be different mechanisms of intervening and creating effects. The linear approach usually focuses on standard tracks or expected treatment responses [12], based on infrequent measures (eg, session by session). The nonlinear frame is based on the conceptualization of psychotherapy as a self-organizing and chaotic process [10,11] and does not presume any assumptions on the shape of trajectories [13]. Presuming standard tracks, pathways of clinical change are expected to run on these tracks, with deviations being at risk of deteriorations or unfavorable developments [14,15]. In case of “not on track” courses, clinical support tools are offered [16]. Different from this, nonlinear monitoring and feedback is based on more frequent and equidistant measures (eg, daily assessments) and, in consequence, on continuous feedback interviewing and process reflection (eg, in every session or every second session). Sampling rates with frequent and equidistant measures allow for different options of analyzing the pathways (time series) of change [17]. No specific incidents (eg, “not on track” states) are required for executing feedback talks. A focus of interest is on occurring critical instabilities, which are valued as precursors of upcoming pattern transitions [18].

Different from high-frequency process monitoring, the so-called routine outcome monitoring [19] focuses on outcome criteria, which are usually given by standard outcome questionnaires such as Outcome Questionnaire-45.2, Symptom Checklist-90-Revised, Depression Anxiety Stress Scales, or Beck Depression Inventory. The sampling rate is low, for example, each month or lower. In consequence, no real time series can be created. In contrast, the process monitoring approach as applied in this study includes a spectrum of common factors that have been proven to be important for creating successful treatments [20]. A Therapy Process Questionnaire (TPQ) was developed for daily self-assessments, which are not restricted to in-session evaluation but can be carried out in the real-world setting of the patients (eco-systemic approach). It includes factors as “well-being and positive emotions,” “emotional and problem intensity,” “motivation for change,” “insight/confidence/therapeutic progress,” and “mindfulness/self-care” [21].

Another difference exists between standardized and personalized procedures, with standardized approaches using predefined questionnaires, whereas personalized measures usually are based on case formulations as the idiographic system modeling and can be implemented by a questionnaire editor, as it is available in the synergetic navigation system (SNS) [22]. As practical experience shows, compliance rates can be optimized by personalized approaches of monitoring and feedback because they focus on patients’ own goals and topics [23]. The items of the questionnaires in use are created by the patients themselves, improving the experienced meaningfulness and thereby, the validity of the measures.

In consequence, there may be different mechanisms of creating effects and benefits by different approaches. Complex high-frequency procedures need intense training for the professionals, closer contact with the patients, a higher frequency of feedback sessions, and a higher degree of personal involvement of the patients, with corresponding effects on emotions, self-experience, and behavior by daily self-assessments (“measurement is intervention”). Clinicians also report on different attitudes to critical periods (instabilities) which are more desirable and less threatening if you see psychotherapy as a self-organizing process running through cascades of pattern transitions, and open-mindedness to different patterns of change. Other mechanisms may be more similar between different approaches, for example, that feedback may provide new information (though it may be various types of information that seem to be important), that feedback enhances patient-clinician communication (though it may focus on different topics), or that it may improve the therapeutic alliance [24]. Successful strategies of implementation into the respective setting of application, training procedures with different contents and messages concerning linear or nonlinear processes, and the clinician’s attitude toward the usefulness of the procedure seem to be important [4].

The research question of the study presented in this paper focuses on the effects of personalized high-frequency feedback based on case formulation (idiographic system modeling) in the context of a nonlinear dynamic systems approach combined with a hypno-psychotherapy called autosystemic hypnotherapy (ASH; original German term: Autosystemhypnose), compared with treatment as usual, in this case, ASH only.

### Hypnotherapy and the Concept of ASH

Hypnotherapy is an evidence-based treatment method that has gained increasing recognition in psychological and medical settings. Its effectiveness is particularly well established in the treatment of anxiety disorders (ADs): a meta-analysis of 17 studies (N=690) [25] reported medium to large effect sizes, showing that patients who received hypnosis for anxiety demonstrated a weighted mean effect size of 0.79 (95% CI 0.61-0.97) posttreatment, suggesting that the average hypnosis patient improved by more than 79% (based on Cohen *d* interpretation) compared to control participants. At follow-up (7 studies, N=215), the mean effect size was 0.99 (95% CI 0.74-1.24), reflecting an improvement greater than 84% of controls. Hypnotherapy has also proven highly effective for depression. In an RCT [26], hypnotherapy was found to be noninferior to cognitive behavioral therapy in treating mild to moderate depression. After 12 months, the response rate (defined as ≥50% symptom reduction) was 44.6% (33/74) of patients who responded to hypnotherapy (compared to 30/78, 38.5%, for cognitive behavioral therapy), indicating sustained therapeutic effects.

The therapeutic efficacy of hypnotherapy is based on its structured therapeutic process and its neurobiologically grounded mechanisms. Hypnotherapy typically follows four evidence-based phases [27]:

- Prehypnotic preparation: therapist and patient explore beliefs, previous experiences, and treatment goals (eg, “reducing panic attacks from daily to ≤1 per week”) while clarifying misconceptions about hypnosis. This builds trust and aligns expectations.
- Hypnotic induction: using focusing techniques or verbal guidance, the therapist induces a state of focused and relaxed attention. Critical reasoning is bypassed, and a trance state is typically reached within 5-10 minutes.
- Therapeutic suggestions: this phase involves tailored therapeutic suggestions and metaphors designed to elicit emotional, psychological, or physiological changes aligned with the treatment goals. These use the brain’s heightened suggestibility during trance to reduce symptoms and promote adaptive experiences.
- Posthypnotic integration: before returning to normal awareness, patients receive posthypnotic suggestions to reinforce and integrate change into daily life. They are encouraged to apply trance-derived insights to real-world situations.

Recent neuroimaging research has provided compelling evidence that hypnosis induces distinct and measurable changes in brain activity. Using multimodal imaging, the research team [28] demonstrated that neural state changes occur synchronously with specific hypnotic induction instructions, indicating that hypnosis produces targeted modulations of brain function rather than nonspecific relaxation effects. In clinical populations, hypnosis has been shown to markedly inhibit the neural response of fear-related circuits, supporting its therapeutic efficacy in anxiety regulation:

- Amygdala inhibition: functional magnetic resonance imaging studies show reduced activity in fear-processing centers during anxiety-focused hypnotherapy, correlating with symptom improvement.
- Suppression of the default mode network: trance reduces self-referential thought, helping patients disengage from rumination patterns.
- Enhanced top-down regulation: increased connectivity between prefrontal and limbic regions allows for improved cognitive restructuring of emotional responses.

This dual foundation—systematic procedural structure and targeted neuroplasticity—explains hypnotherapy’s superiority over placebo in meta-analyses [25,26] and supports its role as a method for modulating emotional and cognitive processes through attentional and self-regulatory mechanisms.

ASH [29] is a variant of hypnotherapy that integrates self-organizing systems theory and synergetic principles with classical hypnotherapeutic elements. As in traditional hypnotherapy, the therapist uses induction and suggestion, but ASH specifically addresses the client’s internal “autosystem” (self-regulatory system) and supports the development of self-observation and resource orientation. While ASH incorporates the established components of hypnotherapy (induction, suggestion, and integration), it adds additional distinctive features:

- Diagnostic precision through hypnotic analysis: virtual “system scans” during trance enable the identification of unconscious blocks (eg, a patient visualizing their depression as “a black hole sucking away motivation” reveals implicit helplessness schemas), as well as exploration of the adaptive function of symptoms (eg, chronic pain maintaining caregiver attention).
- Resolution of multilayered resistances: the therapist guides the patient from addressing superficial objections (“therapy takes too long”) toward deeper levels of processing in which core conflicts (“if I recover, my partner will leave me”) are uncovered and reframed through trance-based cognitive restructuring.

While ASH has an established theoretical foundation and clinical tradition in German-speaking countries [30], no controlled efficacy studies exist to date. Accordingly, the present project aims to evaluate the effectiveness of ASH within a controlled design, building upon the broader evidence base for hypnotherapy.

### Major Depression and ADs

The planned study will apply feedback-informed psychotherapy in an outpatient setting to patients with major depressive disorder (MDD) and the spectrum of ADs, including MDD with anxiousness disorders. MDD is one of the most important mental health issues all over the world. It affects about 3.8% of the global population, or about 280 million people of all ages, with an increasing trend [31]. MDD is about 50% more common among women than among men. More than 700,000 people die due to depression-related suicide every year. MDD is characterized by a mental state of low mood and aversion to activity. It affects the thoughts, behavior, feelings, and sense of well-being of the person concerned. Experiences that would normally bring a person pleasure or joy give reduced pleasure or joy, and the afflicted person often experiences a loss of motivation or interest in many different activities. It may feature sadness, difficulty in thinking and concentration, or a significant increase or decrease in appetite and time spent sleeping. People experiencing MDD may have feelings of dejection or hopelessness and may experience suicidal thoughts. MDD is one of the most common comorbidities of many chronic diseases, including cancer and cardiovascular, metabolic, inflammatory, and neurological disorders. It is also a frequent comorbidity of other mental disorders, such as AD, borderline personality disorder, or obsessive-compulsive disorder. AD is the world’s most common mental disorder, affecting 301 million people in 2019 [32]. People with an AD may experience excessive fear or worry about a specific situation (for example, a panic attack or social situation) or, in the case of generalized anxiety disorder (GAD), about a broad range of everyday situations. They typically experience these symptoms over an extended period, at least several months. Usually, they avoid situations that make them anxious. Other symptoms of AD may include trouble concentrating or making decisions, feeling irritable, tense, or restless, experiencing nausea or abdominal distress, heart palpitations, sweating, trembling or shaking, trouble sleeping, or a sense of impending danger, panic, or doom. AD increases the risk for depression and substance use disorders, as well as the risk of suicidal thoughts and behaviors.

There are different kinds of AD, including GAD, characterized by persistent and excessive worry about daily activities or events, panic disorder, social AD, specific phobias, and others. Psychological mechanisms of GAD illustrate the comorbidity between anxiety and depression, for example, uncontrollable worry, constituting maladaptive strategies such as avoiding anxiety-related situations and emotional states. Individuals enduring GAD show deficits in detecting and regulating emotional states, which might accelerate a positive feedback loop between general stress symptoms and pathological worrying [33]. Experiential avoidance might lead to a restriction in proactive behavior because individuals will be focused on preventing negative events and maintaining safety [34].

### Therapist Effects in Randomized Clinical Trials

In this study, a randomized allocation of the treatment conditions will be realized among the therapists. Although blinded allocation of patients and health professionals is an important claim in double-blind randomized pharmacological trials, in evidence-based human interventions, for example, psychotherapy, health professionals cannot be blinded, because hopefully they are well-informed and fully aware of the interventions they apply [20,35]. This implies that personal preferences and the degree of identification with the treatment procedures that are conducted by the therapist (so-called allegiance effects) may affect the quality of the treatment as well as the outcome. However, therapist effects seem to be an important general (nonspecific) factor, which also will have consequences for the quality of processes and outcomes. Research on common factors revealed that therapist effects have bigger effects than treatment differences, in naturalistic designs, but even in RCTs, which usually are designed to minimize therapist effects (eg, by implementing treatment protocols) [20,36]. In consequence, crossed-therapist designs were suggested for naturalistic intervention studies [37,38], where a therapist is allocated to two or more treatment conditions, and therefore, potential differences in overall therapist effectiveness can be estimated across conditions. To estimate therapists’ personal treatment preferences, which may impact their effectiveness [8,39], these preferences should explicitly be assessed [20,38,40].

### Aims of This Study

This study addresses different research questions. There is some concern regarding the implementation of the procedure into the routine practice of outpatient psychotherapists, which is challenging because of the complexity of the routine process monitoring procedure. It encompasses an assessment of the patient’s personal resources, a case formulation, and, based on this, the development of a personalized process questionnaire. Regular feedback interviews during the sessions are scheduled with reference to the visualized results. Other research questions focus on the outcome of this study’s conditions and on the patterns of change that may characterize successful or unsuccessful therapies. Potential therapists’ implementation effects will be examined based on an ABAB therapist allocation (see the Study Design section). The main research questions are as follows:

1. Practicability: Can high-frequency process monitoring (daily self-assessments) and feedback interviewing (at least every second session) be implemented in outpatient routine psychotherapy, that is, in naturalistic settings? To what extent is the routine practice of outpatient psychotherapy supported by process monitoring? What can be learned from this study for the implementation of process monitoring into practice and training?
2. Treatment outcomes: What is the efficacy of ASH compared with a waiting period control? What are the differences in the treatment efficacy of the two randomized implementation conditions, ASH versus ASH supported by personalized process monitoring and feedback in self-reported primary and secondary outcomes?
3. Session effects and change dynamics: What are the differences between the implemented conditions in the experienced quality of the sessions (eg, micro-outcome) and in the therapeutic alliance? Does the process feedback support the therapeutic alliance? With reference to the time series created by the daily self-assessments: Are there specific features of the change dynamics (eg, discontinuous pattern transitions and critical instabilities) related to the outcome?
4. Therapist effects: Are there any differences across therapists (therapist effects)? Is there an interaction between potential therapists’ preferences for a particular implementation condition and outcome?

## Methods

### Study Design

This study is designed as an RCT with 2 arms, using within-therapist randomization in the real-world clinical practice of routine outpatient psychotherapy in Germany.

Participants are recruited offline through routine intake procedures when presenting for outpatient psychotherapy with participating therapists. Patients who are suspected by the therapist to meet criteria for MDD, AD, or MDD with anxious disorders complex will be briefly screened for their ability and willingness to use a smartphone. If eligible, they will be informed about this study and its requirements by the respective therapist and asked whether they are interested in participating in a research project. Afterward, they will be informed of the procedure and the requirements of this study by the respective therapist. They obtain a flyer with basic information and, in case of concrete interest, will be instructed in detail and obtain an explanation to its full extent.

Patients who have consented to participate in this study will undergo a structured diagnostic interview (Structured Clinical Interview for *DSM-5* [*Diagnostic and Statistical Manual of Mental Disorders* {Fifth Edition}] – Clinician Version) [41] to confirm the inclusion criteria (diagnoses as listed in the Participants section, see below). The interview will be conducted remotely by an independent, trained clinical psychologist who is otherwise not involved in this study. The Structured Clinical Interview for *DSM-5* allows a final decision on including or excluding the patient. In addition to diagnostic confirmation, baseline information on participants’ current medication, concurrent treatment, and symptom severity will be systematically assessed. These variables will be considered as covariates in the main outcome analyses to account for potential prognostic influences. In addition, sensitivity analyses will be conducted to examine whether excluding participants with concurrent treatments or psychopharmacological medication changes affects the robustness of the findings. Following inclusion, a waiting period of 1 (minimum) to 6 months (maximum) begins for each patient, depending on the therapist’s capacity to initiate treatment.

After the waiting period, a randomization procedure decides on the allocation of a patient to condition A (ASH) or B (ASH+synergetic process management [SPM]). To ensure methodological rigor and internal validity, patients will be randomly assigned to one of the two treatment conditions using therapist-specific block randomization. The predefined block-randomized allocation sequences (eg, ABAB, BABA, AABB, BBAA, ABBA, and BAAB) will be generated in advance by the principal investigator for each therapist to ensure a balanced distribution of conditions within therapists. Patients will be assigned to intervention conditions in the order of study inclusion according to the next position in the respective therapist’s allocation sequence. The allocation sequence will remain concealed from the therapists until each new patient is assigned.

After the randomization, the treatment period starts, whose length depends on the concrete requirements of each patient and must be decided by the patient and therapist together (shared decision-making). In contrast to manualized treatments (adherence to protocol), this is a usual procedure in naturalistic settings. A follow-up assessment will be conducted 6 months after the end of therapy.

**Figure 1:**
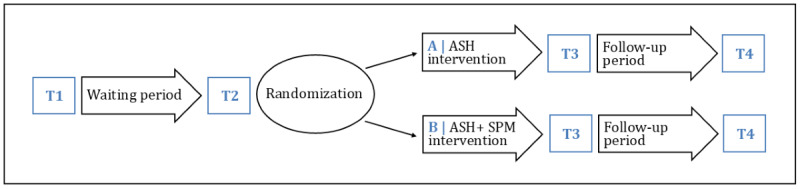
Design of this study. ASH: autosystemic hypnotherapy; SPM: synergetic process management; T1: beginning of the waiting period; T2: at the start of the treatment; T3: at the end of the treatment; T4: at follow-up.

There are four assessment points: at the beginning of the waiting period (T1), at the start of the treatment (T2), at the end of the treatment (T3), and at follow-up (T4). The design of this study is illustrated in Figure 1.

### Ethical Considerations

This study received ethical approval from the Ethics Committee of the Faculty of Psychology and Educational Sciences at Ludwig Maximilian University, Munich (approval number: not specified in protocol; approval letter dated November 8, 2021). The use of the SNS was additionally approved by the Ethics Committee of the Province of Salzburg (No. 415-E/1068/3–2009).

All participants provided written informed consent before the intake assessment (T1). The consent form detailed the study’s purpose, procedures, potential benefits/risks, and data anonymization. Participants were explicitly informed that (1) participation is voluntary and may be withdrawn at any time without consequence, (2) declining or withdrawing imposes no detriment to their clinical care, and (3) no financial compensation or gratuities are offered.

All collected data are subject to data protection regulations and will be published in anonymized form, ensuring participant confidentiality. These procedures are in accordance with the Declaration of Helsinki [42].

### Participants

As this study involves self-assessments via a smartphone application of the SNS, participants are required to have basic smartphone and internet literacy. This is assessed informally during the initial instruction and onboarding process by the respective therapist.

The patients who will be included in this study should be diagnosed with a spectrum of depression and ADs as listed in the *ICD-10* (*International Statistical Classification of Diseases, Tenth Revision*) [43], which is still valid in Germany. These are F32 depressive episode, F33 recurrent depressive disorder, F34 persistent mood disorders, F40 phobic ADs (eg, agoraphobia, social phobia, and specific phobias), F41 other ADs (eg, panic disorder, GAD, mixed anxiety and depressive disorder), F43 reaction to severe stress and adjustment disorders, and F45 somatoform disorders. At least one of these categories should be identified as the main diagnosis; other diagnoses (comorbidities) will be accepted. The reason for focusing on these diagnoses is their high prevalence rates and in consequently, the frequency of occurrence in routine outpatient psychotherapy. Exclusion criteria are aged younger than 18 years, acute suicidality, mental confusion, intoxication by drugs, substance use, and severe neurological diseases.

### Treatment Protocol

This study comprises 2 conditions. One is ASH, which is a version of existing hypno-psychotherapy. It comprises exercises in imagination, activating inner parts (ego-states), and training in mentalization and relaxation. Exercises should induce altered states of consciousness, and triggered ideomotor movements should allow some direct “communication” with “the unconscious.” Resources are activated by the imagination of symbolic persons, figures, or other objects, which might help to protect or support the person to realize changes in their life, to prepare decisions, or to accept and overcome traumatic experiences.

The other condition of this study combines ASH with an advanced method of personalized routine process monitoring, the SPM [44]. The SPM includes (1) an interview for activating personal resources and by this, induces a mental resource state; (2) a method of case conceptualization, which creates a qualitative network model of the most important psychological and social conditions (variables) of a problem [45,46]; and from this, (3) develops a personalized process questionnaire (each item of the questionnaire represents a variable of the model) in close cooperation between the patient and the therapist.

The monitoring is realized by the internet- and server-based device SNS, which collects, stores, analyzes, and visualizes the incoming data from the patient’s smartphone [44]. Patients access the SNS as part of their therapeutic process, supported by their therapist, and use the app in their daily life outside the therapy sessions (ecological setting). No payment is required, and internet access is assumed through patients’ own smartphones. Patients complete one self-assessment per day via the SNS app. They can activate optional reminder notifications in the SNS app to support adherence to daily self-assessments. However, there are no additional prompts such as emails, SMS text messaging, or phone calls from this study’s team.

**Figure 2:**
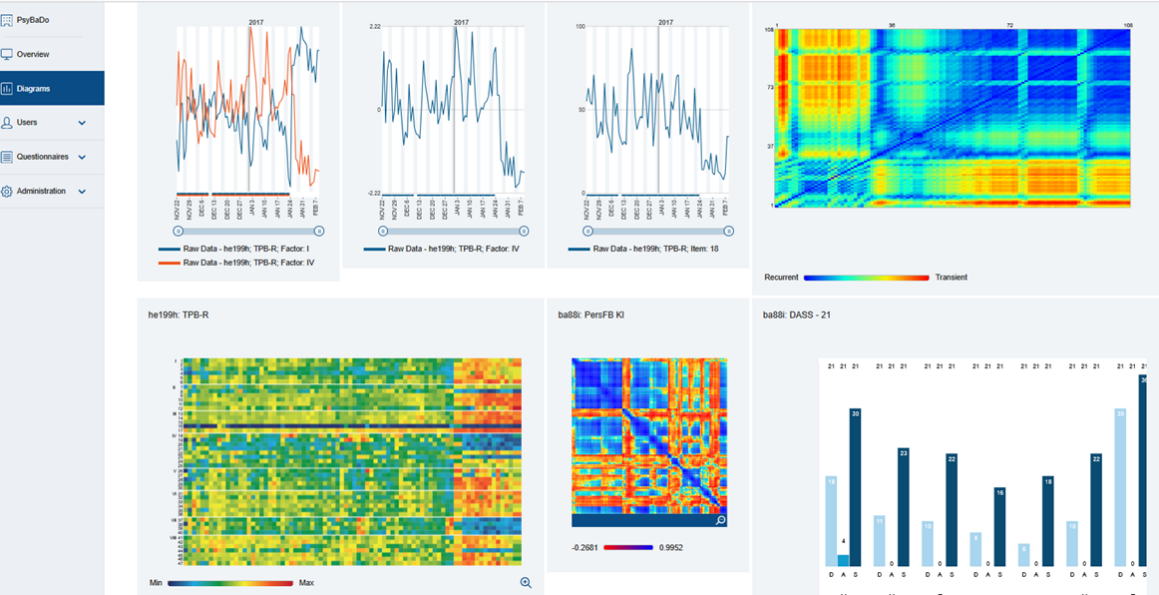
Screenshot of the visualized data in the device SNS. DASS-21: Depression Anxiety Stress Scales – 21 Items; SNS: synergetic navigation system; TPB-R: Therapy Process Questionnaire (German: Therapie-Prozessbogen-Revidiert).

A final but important part of the SPM is (4) a regular feedback interview on the visualized data of the ongoing process. The feedback interviews take place in standard face-to-face therapy sessions. It can be based on several heuristics, which define important conditions for self-organized pattern transitions to be realized during the change process of a patient, the so-called “generic principles” [44]. The design and functionality of SPM and the SNS are based on the theoretical framework of nonlinear dynamic systems theory applied to psychotherapy. This approach conceptualizes therapeutic change as a self-organizing process characterized by dynamic instabilities, pattern transitions, and emergent reorganization. In the SNS, this framework is operationalized by the continuous self-assessment of individualized process variables, visualized as time series (see Figure 2 for a screenshot of the visualized data in SNS). The system does not rely on standardized change trajectories but allows for the identification of nonlinear phenomena such as phase transitions, critical fluctuations, and synchronization across process dimensions. These visualized patterns are reflected upon during regular feedback interviews and serve as the basis for process-oriented decision-making in therapy. The feedback is thus not triggered by deviations from a normative path (eg, “not on track”) but by the recognition of system-dynamic signals emerging from the individual course of therapy. A recent case study illustrates how ASH with SPM can be integrated [47].

In the ASH+SPM condition, the personalized questionnaire of each patient will be combined with the standardized and validated TPQ [21], both administered at the same sampling rate (once per day).

### Implementation Check

At the end of each session, therapists will estimate to what extent the features and criteria of the respective treatment condition were implemented in the current session. For this purpose, a list of criteria was compiled for each condition. Examples of the ASH criteria are “in this session we applied methods from ASH, like analysis of ambiguity and resistance,” “in this session we activated inner parts (problem or solution-related parts) and archetypes,” “in this session, we used symbolizations,” “in this session, we used metaphors,” “in this session, I supported the patient to apply autosystemic hypnotherapy techniques autonomously.” Examples of the SPM list are “in this session, we reflected the process by using its visualization in the SNS,” “this session was planned and realized with reference to the generic principles,” “in this session, the resources of the patient were important (eg, by conducting a resource interview),” or “in this session, we created a system model or referred to this.” Response options range from “not at all” to “very clearly.”

Beyond these kinds of session ratings by the therapists, regular supervision meetings will be offered to all therapists. These meetings are intended to support the therapists in their work, to reflect on the ongoing process, for example, on difficult situations during the treatments, such as lack of progress and breaks in the alliance, and to obtain an impression from a third-party point of view on which the criteria of the treatment conditions were fulfilled.

### Therapists

All therapists to be included are trained and experienced in ASH, with a professional background in medicine or psychology. All are approbated physicians or psychotherapists according to the German psychotherapy law, with at least some years of practical experience in outpatient psychotherapy. For acquiring competencies in SPM, 8 online training modules on the components of the SPM will be performed, particularly idiographic system modeling and case conceptualization, developing personalized process questionnaires, feedback interviewing, technical handling of the SNS, and understanding and interpreting the diagrams as presented by the SNS. At least 15 therapists will participate in this study, each delivering both conditions.

### Assessments

To evaluate treatment effectiveness, both primary and secondary outcome measures will be assessed at all four measurement points (T1-T4), with participants completing the assessments via online questionnaires in the SNS app outside the therapy sessions.

Primary outcomes focus on symptom reduction in depression and ADs and will be measured using (1) the short version of the Symptom Checklist – 14 Items [48]; and (2) the Depression Anxiety Stress Scales – 21 Items, short version [49,50].

Secondary outcomes include measures of psychosocial functioning, emotion regulation, and quality of life: (1) the General Self-Efficacy Scale [51], (2) the Intercultural Quality of Life Comic [52], and (3) the Difficulties in Emotion Regulation Scale [53,54].

Process and moderator variables will be collected throughout the intervention.

At T2, the motivation for psychotherapy will be measured via a psychotherapy motivation questionnaire (Fragebogen zur Psychotherapiemotivation – 23 Items, FPTM-23) [55] to check for bias between treatment conditions. After each therapy session, the Bern Patient and Therapist Session Questionnaire [56] will assess therapeutic alliance and session quality (therapist and patient reports).

By this, potentially important mediating and moderating variables of the treatment conditions should be incorporated in the analysis, for example, the course of the alliance, the session-by-session micro-outcome, motivation for change, activation of resources, and others.

Additionally, the tool SNS will be used for personalized feedback and monitoring in the ASH+SPM condition, enabling daily assessment of intraindividual change processes based on the TPQ for outpatient psychotherapy [21] in combination with a personalized process questionnaire developed individually with each patient.

### Power Considerations

The estimation of the sample size by power analysis refers to two research questions: (1) Is there a difference between the waiting period and the treatment? (2) Is there a difference between the two conditions of the treatment? The statistical procedure for answering these questions is ANOVA with repeated measures, with the groups (conditions) as the between-subject factor and the comparison of the waiting period (T1-T2, level 1) with the overall treatment (T2-T3, level 2) as the within-subject factor. The power calculation for estimating the sample size was realized by the software package G*Power (Heinrich-Heine-Universität Düsseldorf) [57].

The power estimation of the within-subjects calculation (waiting period versus overall treatment, independent of the condition) was based on the reported average effect size of psychotherapy outcomes from studies included in the meta-analysis (Cohen *d*=0.68) [58]. Using an effect size converter, this d value was transformed into Cohen f (f=0.34), which is needed for calculations in G*Power. The power was defined at 0.95, and the correlation between the measurement points was left at a default value of *r*=0.50. Sphericity was assumed. For two measurement points within the sample, it was estimated that the null hypothesis of no difference (no change) could be rejected with a probability of 96% if a sample size of 32 patients is included.

For the power calculation on the effects of the conditions (between-group comparison), a lower threshold value of the mean effect size was supposed at f=0.25, because of the lack of data in this case. The power was defined at 0.80. All other values and assumptions were the same for the between-subject factor as for the intersubject factor. It was estimated that the null hypothesis of no difference (no change) could be rejected with a probability of 80% if a sample size of 98 patients (49 in each condition) were included.

Based on the estimated sample sizes of 32 patients (hypothesis 1) and 98 patients (hypothesis 2), we defined the overall sample size of this study as 100 patients (n=50 in condition A: ASH; n=50 in condition B: ASH+SPM). The allocation ratio is 1:1 with 50 patients per arm.

### Statistical Analysis

The statistical procedure for answering the outcome-related questions of this study is ANOVA with repeated measures, with the groups (conditions) as the between-subject factor and the comparison of the waiting period (T1-T2, level 1) with the overall treatment (T2-T3, level 2) as the within-subject factor. The preference ratings of the therapists in the ASH+SPM condition can be used as covariates in a 1-factor ANOVA with repeated measures.

The therapy effects can be predicted by linear regression models and multilevel regression models with conditions, therapy motivation (patients), alliance rates (patients), and the quality of the implementation as predictors. In the ASH+SPM condition, the preference of implementing the SPM (therapists) and the compliance rates (patients) of the process monitoring can also be used as predictors.

The research questions on the dynamics of change (eg, Are pattern transitions related to the outcome?) will be analyzed by applying the Pattern Transition Detection Algorithm [59] and methods of recurrence quantification analysis [60,61]. The respective quantifiers will be correlated with the outcome measures (T2-T3 and T2-T4).

## Results

Although the trial is ongoing, recruitment has already begun. As of the time of submission, 22 participants have been enrolled and have completed the diagnostic eligibility assessment. Data collection started in 2022 following the training of participating therapists in the use of the SNS, which was funded by the German Society for Autosystemic Hypnotherapy in 2022 and 2025. Data collection will continue until all follow-up assessments (T4) are completed, which is projected for the first quarter of 2027. No outcome analyses have been conducted to date. Study findings are expected to be disseminated in the second quarter of 2027.

## Discussion

### Principal Findings

This study tries to combine the strictness of an RCT with the ecological validity of routine care (outpatient settings). The aims of this study focus on the effectiveness of ASH and of ASH improved by personalized high-frequency monitoring and feedback, which was technologically implemented by the SNS. This kind of comparison has never been conducted in an RCT under real-world conditions, and the combination of outcome and process research from the perspective of nonlinear complex systems [10,62] is an innovative step in psychotherapy research.

Besides the frequency of patients’ self-assessments, the frequency and quality of feedback sessions (participative interviewing and shared decision-making) also matter. Studies have shown that information from feedback is often not used in clinical practice. Although routine outcome monitoring is mandatory in many countries, measuring outcomes (or even processes) does not necessarily mean that this information is being used to inform or to advance the treatment. Even in RCTs, up to half of the clinicians do not use progress feedback to inform treatment decisions [8,63]. In a specific RCT [64], one third of the clinicians reportedly never viewed any progress feedback reports. In a content analysis of written notes [65], clinicians included in the study incorporated feedback data in only about one-fifth of the sessions. A review addressing the barriers to implementing precision methods as feedback in personalizing psychological therapies, and possible solutions, has been published recently [66]. This study investigates an optimal way of implementing feedback in clinical practice (outpatient therapy) and integrating it into treatment as usual.

The combined structure of this study’s design should allow differentiation between patient effects, therapist effects, and different degrees of preferences for the feedback method (SPM). In contrast to pharmaceutical trials, patients and therapists know what they should do and are proactively involved in the treatment. This involvement is not a bias that has to be eliminated; it might rather be an active ingredient of a successful psychotherapy in which the therapists and the patients take a proactive and collaborative role in the treatment plan.

### Bias Minimization

At the level of patients and therapists, biases should be minimized. Patients will be randomly assigned to conditions to reduce systematic selection biases in participant characteristics. The inclusion and exclusion criteria allow for a relatively homogeneous group of individuals with depression-anxiety spectrum diagnoses, and by measuring the psychotherapy motivation at the beginning of the treatment period (T2), motivation biases can be considered (statistically balanced). At the therapist level, a potential bias due to the therapist’s preferences is a concern because of outcome expectations, which can be impactful for patients and for therapists. In the proposed design, potential therapist effects can be considered (statistically balanced), and the present ABAB crossed therapist design can consider the preferences of realizing the SPM procedure.

### Limitations

Despite all the measures taken to optimize the design and minimize biases, there are some limitations due to the naturalistic setting of this study. On the one hand, there are differences in the waiting time, which can be statistically taken into account as covariates in the ANOVA, but cannot be eliminated. The same applies to therapist effects, which can be regarded as a confounding factor, but are not completely eliminated. One reason for this is that it is not yet clear which psychological mechanisms are behind the effects of the therapists, which are more important even in controlled studies than the differences between the treatment conditions [20,36]. Another limitation concerns the implementation check, which is given by self-assessments of the therapists after each session, not by video-based coding of the sessions by independent and blinded raters. Independent session ratings of the quality and the accuracy of the conditions should be realized in future studies, because this can be supposed to have an impact on the outcome, especially in feedback-based therapy sessions.

### Strengths

This study will have a number of strengths. One is the combined process and outcome research, with outcome measures at 4 time points (T1-T4, see the Study Design section) and process monitoring at 2 different sampling rates: session by session with the Bern Patient and Therapist Session Questionnaire in both conditions and high-frequency equidistant time sampling (day by day) in the ASH+SPM condition. Given the 2 sampling rates, the course of micro-outcomes and alliance rates can be aligned (synchronized) with the nonlinear features of change dynamics (eg, critical instabilities, pattern transitions, and within-person synchronization) as they can be derived from the high-frequency time series data. The combination of outcome and process research is still unusual and innovative in RCTs.

Another strength is the strictness of an RCT design implemented in a naturalistic setting, which improves the ecological validity of this study and allows for the transfer to similar settings in outpatient psychotherapy. Third, we will recruit experienced therapists trained in both ASH and SPM (including process monitoring and feedback). As a result, both conditions are performed highly competently and qualify as bona fide therapies. Fourth, we will conduct an implementation review in both study conditions (with the restrictions as noted in the Limitations section). Fifth, in addition to the hypotheses about differential treatment effects (see the Aims of This Study section), mechanisms of change can also be examined.

## Appendix

Multimedia Appendix 1 CONSORT-eHEALTH checklist (V1.6).

Multimedia Appendix 2 Informed consent form.

## Abbreviations

AD: anxiety disorder
ASH: autosystemic hypnotherapy (original German term: Autosystemhypnose)
DSM-5: Diagnostic and Statistical Manual of Mental Disorders [Fifth Edition]
GAD: generalized anxiety disorder
ICD-10: International Statistical Classification of Diseases, Tenth Revision
MDD: major depressive disorder
RCT: randomized controlled trial
SNS: synergetic navigation system
SPM: synergetic process management
TPQ: Therapy Process Questionnaire
