# Supplementary material for: Improving Outpatient Psychotherapy for Adults With Major Depressive and Anxiety Disorders Using Web-Based High-Frequency Monitoring and Feedback in Autosystemic Hypnotherapy: Protocol for a Two-Arm ABAB Crossed-Therapist Randomized Clinical Implementation Trial
Source: JMIR Res Protoc. 2026 Jan 7;15:e78166. doi: 10.2196/78166 (PMC12824570; doi:10.2196/78166)
Supplement: Multimedia Appendix 2 [file resprot_v15i1e78166_app2.pdf]

Ludwig-Maximilians-Universität München
Fakultät für Psychologie und Pädagogik

Forschungsleitung
Prof. Dr. Günter Schiepek
guenter.schiepek@psy.lmu.de

Operative Studienleitung
Stephanie Wackernagel
Psychologin M.Sc.
s.wackernagel@campus.lmu
+49 151 41620226

Datenschutzrechtliche Aufklärung und Einwilligung inkl. Information gem. Art.13 EU-DSGVO entsprechend der Vorgaben der Ethikkommission der Ludwig-Maximilians-Universität München

....
Name der einwilligenden Person in Druckbuchstaben

geboren am ....

Vielen Dank, dass Sie sich für eine Teilnahme an der Studie interessieren. Anbei erhalten Sie Informationen zum Forschungsvorhaben, den Teilnahmebedingungen und zur Handhabung der erhobenen Daten. Bitte lesen Sie alles aufmerksam durch. Wenn Sie einverstanden sind und an der Studie teilnehmen möchten, bestätigen Sie bitte die anschließende Einwilligung.

1. Beschreibung des Forschungsvorhabens

Im Rahmen dieser Studie soll die Wirksamkeit der Autosystemhypnose (ASH) sowie der Mehrgewinn durch das Synergetische Prozessmanagement (SPM, mit täglichen Fragebogenerhebungen über das internetbasierte Synergetische Navigationssystem (SNS), Ressourcenerfassung, individueller Fallkonzeption und Feedbackgespräche) untersucht werden. Dazu werden zwei Behandlungsgruppen realisiert:

A) ASH-Behandlung
B) ASH-Behandlung + Synergetisches Prozessmanagement

Die Zuteilung zu den Behandlungsgruppen erfolgt randomisiert (nach dem Zufallsprinzip) im Anschluss an die Wartezeitphase.

Zur Untersuchung der Wirksamkeit werden die Behandlungsergebnisse der beiden Gruppen in Hinblick auf verschiedene Kriterien (z.B. Symptomreduktion) mit den Ergebnissen der Wartezeit verglichen.

Der zeitliche Umfang der Studie richtet sich nach der individuellen Dauer der psychotherapeutischen Behandlung. Nach einer ausführlichen Information und Aufklärung sowie der Einwilligung in die Studienteilnahme erfolgt eine Wartezeit von ca. 1 - 2 Monaten mit anschließender Behandlungsphase von ca. 3 - 6 Monaten. Vor Beginn der Wartezeit, vor Beginn der Behandlung, zum Behandlungsabschluss und 6 Monate nach Therapieende erfolgt eine umfangreiche Fragebogenerhebung zur Erfassung symptomatischer Belastung, Selbstwirksamkeit, Einschätzung der Lebensqualität und Fähigkeit zur Emotionsregulation. Für die Beantwortung der Fragen sind für die vier Zeitpunkte ca. 45 Minuten einzuplanen. Je nach Gruppenzuteilung (zufällige Zuweisung) erfolgen weitere Fragebogenerhebungen. In der Behandlungsgruppe A (ASH) wird nach jeder Therapiesitzung ein Fragebogen zur Einschätzung der

Therapie erhoben (Berner Patienten-Stundenbogen, Bearbeitungsdauer ca. 15 Minuten). In der Behandlungsgruppe B (ASH+SPM) wird zusätzlich täglich ein Fragebogen, bestehend aus maximal 20 individualisierten Fragen sowie 24 Standardfragen zum Therapie-Prozess (Kurzform des Therapie-Prozess-Fragebogens) erhoben. Hierfür sollten ca. 15 Minuten täglich eingeplant werden. Die Erhebung der Fragebögen erfolgt über einen Patienten-Code (zufällige Buchstaben- und Ziffernfolge), den der/die behandelnde(n) Therapeut*in erstellt. Den Studienleitern ist somit kein Rückschluss auf die Person möglich.

Wie funktioniert das SNS?

Das SNS beruht auf der Erfassung und Darstellung täglicher Selbsteinschätzungen während der Zeit der Therapie. Die Eingabe erfolgt mittels Fragebögen direkt am Computer oder per App. Zusätzlich zum Fragebogen können auch persönliche Kommentare zu aktuellen Ereignissen verfasst werden. Diese persönlichen Kommentare dienen ausschließlich der eigenen Reflexion und können bei Bedarf in den Reflexionsgesprächen mit dem/der Therapeut*in thematisiert werden. Mitteilungen an den/die Therapeut*in können nicht elektronisch, sondern nur persönlich erfolgen. Nach einer individuellen Einführung durch den/die Therapeut*in werden zur täglichen Eingabe ca. 15 bis 20 Minuten benötigt. Auf Grundlage der idiographischen Systemmodellierung (ein Verfahren der Fallkonzeption) wird auch ein persönlicher Prozessfragebogen erstellt, der mit Hilfe des SNS täglich ausgefüllt wird. Die Prozessdaten fließen in die Feedbackgespräche mit dem/der Therapeut*in ein.

Beanspruchung durch die Studie

Die Fragebogenerhebung kann eine vertiefte Selbstreflexion und Beschäftigung mit den eigenen therapierelevanten Themen bedingen, was unter Umständen auch eine Konfrontation mit problematischen Verhaltensmustern und emotionale Belastungen bedeuten kann. Solche Prozesse, ebenso wie andere Veränderungsprozesse, die aus der Therapie resultieren können, sind manchmal herausfordernd. Resultierende Belastungen sollten mit dem/der Therapeut*in zeitnah besprochen werden, um gute Umgangsweisen und Lösungsschritte zu erarbeiten. Es finden keine körperlichen Beanspruchungen statt (wie beispielsweise Blut- oder Speichelentnahme, Medikamenten- oder Placebogabe).

Freiwilligkeit

Die Teilnahme an diesem Forschungsvorhaben ist freiwillig. Die Einwilligung zur Teilnahme kann jederzeit und ohne Angabe von Gründen widerrufen werden, ohne dass daraus Nachteile entstehen. Die Teilnahme an der Studie wird nicht vergütet und geht auch nicht mit andern Vorteilen monetärer oder anderer Art einher. Die Teilnahme ist nicht an andere Dienste oder einen Vertrag gekoppelt. Die Entscheidung an der Studie teilzunehmen (oder nicht), hat keine Vor- oder Nachteile bezüglich der psychotherapeutischen Behandlung. Die Einwilligung kann verweigert und jederzeit zurückgezogen werden, hierdurch entstehen keine Nachteile für die Behandlung.

2. Inhalt und Zweck der Studie

Zweck der Studie ist die Evaluation der Autosystemhypnose (ASH) in Kombination mit oder ohne Synergetisches Prozessmanagement. Dazu werden bei Therapieanmeldung, bei Therapiebeginn, nach jeder Therapiesitzung, bei Therapieabschluss und sechs Monate nach Therapieabschluss Fragebogendaten erhoben. In der Behandlungsgruppe, die das Synergetische Prozessmanagement miteinschließt, werden zudem tägliche Selbsteinschätzungen mit Hilfe des Synergetischen Navigationssystems (SNS) durchgeführt, die der Selbstreflexion und Einschätzung des Therapieverlaufs dienen.

3. Betroffener Personenkreis

An der Studie können volljährige Patient*innen teilnehmen, die eine Autosystemhypnose-Behandlung in Anspruch nehmen möchten und an einer depressiven und/oder angstbezogenen Symptomatik leiden. Eine entsprechende diagnostische Einschätzung erfolgt zu Beginn der Behandlung. Für die Studie nicht geeignet sind Patient*innen mit akuten Verwirrtheitszuständen, akuter Drogenintoxikation, Suchtdiagnose,

akuter Suizidalität oder neurologischen Erkrankungen. Im Verlauf der Studie werden keine Informationen zu weiteren Personenkreisen (z.B. Verwandten) erfragt.

4. Zu erhebende Daten

Im Rahmen der Studie werden neben den Fragebogendaten folgende soziodemografischen Daten erhoben, die der statistischen Auswertung dienen: Alter, Geschlecht, Familienstand, Schulbildung, Beschäftigungsstatus, Diagnose. Die Daten werden mit Hilfe des internetbasierten Synergetischen Navigationssystems (SNS) unter einem Patienten-Code (Pseudonym aus zufälliger Buchstaben- und Zahlenfolge, welches keinen Rückschluss auf die Person ermöglicht) erhoben. Der/die Patient*in kann sich für die Eingabe der Fragebogendaten mit dem Patienten-Code und einem selbst gewählten Passwort über ein Mobil-Gerät (z.B. Smartphone oder Tablet) oder an einem Computer auf der SNS-Internetseite einloggen und die jeweiligen Fragebögen ausfüllen. Ausschließlich dem/der Therapeut*in ist eine Zuordnung der Fragebogendaten zur Person möglich. Es erfolgt keine Weitergabe dieser Daten an Dritte.

5. Analyseergebnisse der Daten

Die wissenschaftlichen Analysen der Studie erfolgen ausschließlich anhand der pseudonymisierten Fragebogen-Daten, wobei die Personen-Zuordnung ausschließlich dem/der behandelnden Therapeut*in möglich ist. Es kann im Rahmen der Studie kein Rückschluss auf die jeweilige Person erfolgen (siehe Punkt 9).

6. Lagerung und Weitergabe von Daten

Verwendete Datenbanken und zugehörige Server

- Datenbankmanagementsystem: Postgres
- Datenverarbeitung und -speicherung im Hintergrund der Anwenderoberfläche: Java Backend
- Betriebssystem: Linux
- Betreiber der serverbasiertes Dienstleistung SNS ist CCSYS GmbH (Prof. Dr. Günter Schiepek). Updates werden ggfs. auf die von der Datenbank getrennte Applikation gespielt
- Die Daten werden auf den Servern des Leibniz-Rechenzentrums der Bayrischen Akademie der Wissenschaften der LMU München gespeichert und dort verarbeitet
- Zugang haben nur die behandelnden Therapeut*innen mit entsprechenden Rechten
- Es werden ausschließlich pseudonymisierte Daten erhoben und gespeichert (Patienten erhalten einen Code)

Komponenten bei Datenlieferanten und Datenabrufenden (Clients)

- Web-Anwendung mit Zugriff via Browser auf die Anwendung
- Patient*innen haben prinzipiell keinen Zugriff auf die Plattform und damit auch keine theoretische Möglichkeit, auf Daten anderer zuzugreifen
- Therapeut*innen haben Zugriff auf die Daten ihrer Patient*innen
- Die Patienten erhalten Zugang über eine in REACT Native entwickelte App, die ausschließlich zur Nutzung als Eingabe-Medium vorgesehen ist

Vernetzungsstruktur zwischen den beteiligten Komponenten

- Anwendung und Datenbanken sind getrennt voneinander in einem Docker-Container innerhalb einer virtual machine in der AWS Cloud Frankfurt und/oder falls gewünscht innerhalb eines VNETS in einem in Deutschland durch SVA gehosteten Microsoft Azure Server.
- Netzwerkinfrastruktur: LAN, separates WLAN, VPN
- Desktop-Clients (Nutzung am PC via): Windows-PC, MAC und Linux-PC
- Mobile Clients (Nutzung an mobilen Geräten via): Windows-Notebooks, Apple-Notebooks, Smartphones und Tablets

Verantwortlichkeit für die Komponenten

- CCSYS GmbH (Prof. Dr. Günter Schiepek)

Pseudonymisierung

Alle für die Studie relevanten Daten werden in pseudonymisierter Form mittels SNS erhoben. Die Pseudonymisierung erfolgt durch den/die behandelnde(n) Therapeut*in. Anhand der erhobenen Daten ist für die Studienleitung kein Rückschluss auf die Person möglich. Weitere Informationen zur Pseudonymisierung siehe Punkt 9.

Vertraulichkeit

- Auf PCs, Laptops, Tablets oder Smartphone der Nutzer (Therapeut*innen) und Administratoren (Studienleitung) liegen keine Daten der Anwendung, erst recht nicht personenbezogene Daten. Die Daten der Anwendung (z.B. beantwortete Fragebögen) liegen auf Servern des Leibniz-Rechenzentrums der Bayrischen Akademie der Wissenschaften der LMU München.
- <u>Zutrittskontrolle:</u> Das Rechenzentrum befindet sich in separaten, abgeschlossenen Räumlichkeiten.
- <u>Zugangskontrolle:</u> Schutz vor unbefugter Systembenutzung durch Kennwörter (einschließlich entsprechender Policy), automatische Sperrmechanismen aufgrund der systemeigenen Sicherheitsrichtlinien des entsprechenden Devices und Sperrung des Users.
- <u>Zugriffskontrolle:</u> Kein unbefugtes Lesen, Kopieren, Verändern oder Entfernen innerhalb des Systems, z.B.: Standard-Berechtigungsprofile auf „need to know-Basis", Standardprozess für Berechtigungsvergabe, Protokollierung von Zugriffen, periodische Überprüfung der vergebenen Berechtigungen, insbesondere von administrativen Benutzerkonten.

Integrität

- <u>Weitergabekontrolle:</u> Kein unbefugtes Lesen, Kopieren, Verändern oder Entfernen bei elektronischer Übertragung durch die direkte Anbindung über Virtual Private Networks (VPN) in das Rechenzentrum.
- <u>Eingabekontrolle:</u> Feststellung, ob und von wem personenbezogene Daten in Datenverarbeitungssysteme eingegeben, verändert oder entfernt worden sind, z.B.: Protokollierung, Dokumentenmanagement.

Verfügbarkeit und Belastbarkeit

- <u>Verfügbarkeitskontrolle:</u> Die Daten liegen auf räumlich getrennten Servern bzw. Clouds. Schutz gegen zufällige oder mutwillige Zerstörung bzw. Verlust durch tägliche Datensicherung auf Bandmedien, unterbrechungsfreie Stromversorgung (USV), Security Checks auf Infrastruktur- und Applikationsebene, mehrstufiges Sicherungskonzept mit Auslagerung zumindest eines Sicherungsbandes.
- Rasche Wiederherstellbarkeit aufgrund Absicherung der Rechenzentrumssysteme über Wartungsverträge.

Verfahren zur regelmäßigen Überprüfung, Bewertung und Evaluierung

- Datenschutz-Management, einschließlich regelmäßiger Mitarbeiter-Schulungen.
- Datenschutzfreundliche Voreinstellungen durch Überprüfung der Benutzerberechtigungsstruktur.

7. Beteiligte, Datenflüsse und speichernde Stellen

Die wissenschaftliche Auswertung der pseudonymisierten Daten wird durchgeführt von:

Prof. Dr. Günter Schiepek
Forschungsleitung der Studie, Zugang zu pseudonymisierten Fragebogen-Daten
Fakultät für Psychologie und Pädagogik
Ludwig-Maximilians-Universität München
Leopoldstr. 13
80802 München
guenter.schiepek@psy.lmu.de

und

Paracelsus Medizinische Privatuniversität (PMU)
Institut für Synergetik und Psychotherapieforschung
Campus Christian-Doppler-Klinik, Haus 38
Ignaz-Harrer-Straße 79
A - 5020 Salzburg

M.Sc. Psych. Stephanie Wackernagel
Operative Studienleitung, Zugang zu pseudonymisierten Fragebogen-Daten
Fakultät für Psychologie und Pädagogik
Ludwig-Maximilians-Universität München
Leopoldstr. 13
80802 München
s.wackernagel@campus.lmu
+49 151 41620226

Marc-Alexander Bäumer
Operative Studienassistenz, Zugang zu pseudonymisierten Fragebogen-Daten
Gräfstraße 113
81241 München
info@heilpraktiker-baeumer.de

Dr. Kathrin Viol
Zugang zu pseudonymisierten Fragebogen-Daten
Paracelsus Medizinische Privatuniversität (PMU)
Institut für Synergetik und Psychotherapieforschung
Campus Christian-Doppler-Klinik, Haus 38
Ignaz-Harrer-Straße 79
A - 5020 Salzburg

Verantwortlicher für die Einhaltung des Datenschutzes:

Prof. Dr. Günter Schiepek
Forschungsleitung der Studie, Zugang zu pseudonymisierten Fragebogen-Daten
Fakultät für Psychologie und Pädagogik
Ludwig-Maximilians-Universität München
Leopoldstr. 13
80802 München
guenter.schiepek@psy.lmu.de

Verwendung anonymisierter Daten

Die Ergebnisse und Originaldaten dieser Studie werden als wissenschaftliche Publikation veröffentlicht. Dies geschieht in vollständig anonymisierter Form, d.h. ohne, dass die Daten den jeweiligen Teilnehmer*innen an der Studie zugeordnet werden können. Die vollständig anonymisierten Daten dieser Studie werden als "open data" in einem sicheren, internetbasierten Repositorium namens Open Science

Framework (https://osf.io/) zugänglich gemacht. Damit folgt diese Studie den Empfehlungen der Deutschen Forschungsgemeinschaft (DFG) zur Qualitätssicherung in Bezug auf Nachprüfbarkeit und Reproduzierbarkeit wissenschaftlicher Ergebnisse, sowie der optimalen Datennachnutzung.

8. Konkrete Dauer der Speicherung

Die Personen-Zuordnungslisten werden von den Therapeut*innen unmittelbar nach Beendigung der Studie gelöscht. Weitere Daten, die im Rahmen der Studie erhoben wurden, werden durch die Studienleitung spätestens 10 Jahre nach Beginn der Datenerhebung gelöscht. Die Primärdaten werden vollständig anonymisiert gespeichert, s. Punkt 7.

9. Pseudonymisierungsverfahren

Die Pseudonymisierung erfolgt durch den/die Therapeut*in in ihrer/seiner psychotherapeutischen Praxis. Nachdem der/die Patient*in zur Teilnahme an der Studie eingewilligt hat, erstellt der/die behandelnde Therapeut*in einen Patienten-Code (zufällige Ziffern- und Buchstabenfolge) aus dem sich der Realname oder sonstige Personenmerkmale nicht rekonstruieren lassen. Die Personen-Zuordnungslisten liegen ausschließlich dem/der behandelnden Therapeut*in in Papierform vor, wird unabhängig von der Patienten-Akte datenschutzgerecht aufbewahrt und an keiner anderen Stelle abgespeichert. Unter dem jeweiligen Code werden die Fragebogendaten dann für die Studie mittels SNS erhoben. Im Rahmen des „digitalen Tagebuchs“ sollen keine spezifischen Angaben (z.B. Ort oder Namen) gemacht werden, sodass kein Rückschluss auf die Person möglich ist. Im Rahmen der Studie ist entsprechend keine Verknüpfung von fragebogen- und personenbezogenen Daten möglich. Anhand der erhobenen Daten ist kein Rückschluss auf die Person möglich.

10. Rechtsgrundlagen

Die Rechtsgrundlage zur Verarbeitung der genannten personenbezogenen Daten bildet die Einwilligung gemäß Art. 6 (1) Buchstabe A EU-DSGVO im zweiten Teil dieses Dokumentes.

11. Widerruf seitens des Betroffenen

Sie haben das Recht, jederzeit die datenschutzrechtliche Einwilligung rückgängig zu machen (zu widerrufen). Durch den Widerruf der Einwilligung wird die Rechtmäßigkeit, der aufgrund der Einwilligung bis zum Widerruf erfolgten Verarbeitung nicht berührt (Widerruf mit Wirkung für die Zukunft). Richten Sie den Widerruf an den Verantwortlichen (nachfolgend). Ihnen entstehen durch den Widerruf keine Nachteile. Nach Eingang des Widerrufs werden die personenbezogenen Daten gelöscht.

12. Namen, Kontaktdaten des Verantwortlichen

Da keine Verarbeitung personenbezogener Daten erfolgt, bedarf es keinen entsprechenden Verantwortlichen.

Die fachliche Verantwortung liegt bei:

Prof. Dr. Günter Schiepek
Fakultät für Psychologie und Pädagogik
Ludwig-Maximilians-Universität München
Leopoldstr. 13
80802 München
guenter.schiepek@psy.lmu.de

und

Paracelsus Medizinische Privatuniversität (PMU)
Institut für Synergetik und Psychotherapieforschung

Campus Christian-Doppler-Klinik, Haus 38
Ignaz-Harrer-Straße 79
A - 5020 Salzburg

13. Kontaktdaten der lokalen Datenschutzkontaktperson des Departments Psychologie

Dr. Nicole Heitzmann
Datenschutzkontaktperson des Departments Psychologie
Ludwig-Maximilians-Universität München
Empirische Pädagogik und Pädagogische Psychologie
Leopoldstr. 13
80802 München
datenschutz.psychologie@psy.lmu.de

14. Hinweis auf Rechte der Betroffenen

Gemäß Art. 13 II b der Datenschutzgrundverordnung haben Sie das Recht auf

Auskunft (Art 15 DSGVO und §34 BDSG)
Widerspruch (Art. 21 DSGVO und §36 BDSG)
Datenübertragbarkeit (Art 20 DSGVO)
Löschung (Art 17 DSGVO und §35 BDSG)
Einschränkung der Verarbeitung (Art 18 DSGVO)
Berichtigung (Art 16 DSGVO)

Möchten Sie eins dieser Rechte in Anspruch nehmen, wenden Sie sich bitte an den behördlichen Datenschutzbeauftragten der Ludwig-Maximilians-Universität München:

Herr Dr. jur. Marco Wehling, LL.M.
Ludwig-Maximilians-Universität München
Behördlicher Datenschutzbeauftragter
Geschwister-Scholl-Platz 1
80539 München
Tel.: +49 89 2180 – 2414
Zum Kontaktformular

Weiterhin haben Sie das Recht, Beschwerde bei der Aufsichtsbehörde einzulegen:

Bayerisches Landesamt für Datenschutzaufsicht (BayLDA)
Promenade 27
91522 Ansbach
Tel: +49 (0) 981 53 1300 poststelle@lda.bayern.de Online-Beschwerdeformular bei Datenschutzverstößen:
https://www.lda.bayern.de/de/beschwerde.html

Hiermit willige ich gemäß Art. 6 (1) Buchstabe a EU-DSGVO freiwillig in die Erhebung und Verarbeitung meiner personenbezogenen Daten ein. Ich bin ausreichend informiert worden und hatte die Möglichkeit, Fragen zu stellen. Über die Folgen eines Widerrufs der datenschutzrechtlichen Einwilligung bin ich aufgeklärt worden.

Die schriftliche Aufklärung und Einwilligung habe ich erhalten.

________________ ____________________________________

Datum Unterschrift Patient*in
